# Anti-dsDNA Antibodies are one of the many autoantibodies in systemic lupus erythematosus

**DOI:** 10.12688/f1000research.6875.1

**Published:** 2015-10-01

**Authors:** Shu Man Fu, Chao Dai, Zhenhuan Zhao, Felicia Gaskin

**Affiliations:** 1 Division of Rheumatology, University of Virginia, Box 800133, Charlottesville, VA, 22908-0133, USA; 2 Center for Immunity, Inflammation and Regenerative Medicine, Department of Medicine, University of Virginia, Box 800133, Charlottesville, VA, 22908-0133, USA; 3 Department of Microbiology, Immunology and Cancer Biology, School of Medicine, University of Virginia, Charlottesville, VA, 22908, USA; 4 Department of Psychiatry and Neurobehavioral Sciences, School of Medicine, University of Virginia, Charlottesville, VA, 22908, USA

**Keywords:** Systemic lupus erythematosus, anti-dsDNA antibodies, autoantibodies, lupus nephritis

## Abstract

Anti-dsDNA antibodies are the most studied antibodies of the lupus-related autoantibodies. The dogma is that these are the most important autoantibodies in systemic lupus erythematosus. In this review, evidence is presented to show that these antibodies (as measured by modern clinical laboratories) are not the most important autoantibodies in the diagnosis of systemic lupus erythematosus, and are of limited value in clinical correlation and in predicting disease flares. In addition, they are not likely to be the initiating autoantibodies in lupus nephritis. Thus, several pervasively held beliefs on anti-dsDNA antibodies are not valid. We suggest that anti-dsDNA antibodies should be considered as just one of the many autoantibodies associated with systemic lupus erythematosus.

## Introduction

Systemic lupus erythematosus (SLE) is a syndrome affecting multiple organs with circulating autoantibodies of complex specificities ^1^. Of the SLE-related autoantibodies, anti-dsDNA antibodies have received the most intense investigation. These antibodies have been cited to be specific for SLE and are the antibodies that initiate lupus glomerulonephritis. They are often thought to be of value in correlating with disease activity and predicting flares in SLE. The clinical significance of anti-dsDNA antibodies has been reviewed at the 50 ^th^ anniversary for the description of anti-dsDNA antibodies in SLE ^2^. In view of several recent publications on this topic, this review intends to provide evidence that anti-dsDNA antibodies should be treated as just one of the SLE-related autoantibodies with limited diagnostic and prognostic values in SLE.

## Historic perspective

In order to understand the origin of the myths associated with anti-dsDNA antibodies, it is important to review briefly the history of anti-dsDNA antibodies in their association with SLE. Fifty-eight years ago, Holman and Kunkel ^3^ reported in the July 29, 1957 issue of Science that deoxyribonuclease destroys the antigenic determinant in nucleoprotein that participates in the LE (lupus erythematosus) cell phenomenon, implicating anti-DNA antibodies in the sera of SLE patients. Subsequent to this publication, additional reports appeared to establish that circulating anti-DNA antibodies were present in patients with SLE ^4– 7^. Interest in anti-dsDNA antibodies increased after the publication of the paper by Koffler *et al.* ^8^ that described the elution of anti-dsDNA antibodies from the kidneys of SLE patients with nephritis. Lupus nephritis was considered to be a prototype of immune complex nephritis in man ^9^. Although it was emphasized that other antibody-antigens are likely to be present in lupus nephritis, it was stressed that the anti-dsDNA antibodies-dsDNA system is of paramount importance ^10^.

The importance of anti-dsDNA antibodies in the clinical care of SLE patients has been emphasized in the past. In a remarkable paper by Tan *et al.* ^11^, anti-DNA antibodies were detected in certain lupus patients prior to the onset of severe proteinuria. These antibodies were not detectable during the acute phase of lupus nephritis. Instead, circulating DNA was detected, suggesting antibody excess. Anti-DNA antibodies were measured in this study by immunodiffusion. The immunodiffusion technique measures precipitating antibodies that may have a higher binding affinity to dsDNA. Some aspects of this study were confirmed by the investigation of Hughes *et al.* ^12^. Hughes *et al.* showed that precipitating antibodies rapidly disappeared with therapy and clinical improvement, and patients with the most severe renal disease with complement consumption and renal impairment gave the strongest precipitin lines. Since the immunodiffusion method for the detection of anti-DNA antibodies is no longer used in clinical laboratories, it should be emphasized that immunodiffusion for the analysis of anti-DNA antibodies identifies patients with anti-dsDNA antibodies of higher affinity and concentration. It is also of historical interest to note that Hughes *et al.* ^12^ showed that the Farr technique is more sensitive and less specific, requiring the setting of a binding level (in their case, 20%) to make the assay specific for SLE.

Since these early publications, there have been many published papers to support the importance of anti-dsDNA antibodies as a biomarker in the diagnosis, pathogenesis, and prognosis of SLE (reviewed in 2, 13, and 14).

## Anti-dsDNA antibodies are not specific or the best biomarker for SLE

Anti-dsDNA antibodies have been included in the 1982 American College of Rheumatology (ACR) revised criteria for the classification of SLE and in the 1997 update of the criteria for the classification of SLE ^15, 16^. The 10 ^th^ criterion includes “antibody to native DNA in abnormal titer”. Edworthy *et al.* ^17^ used the Stanford Lupus Cohort (n=339) and matched controls to validate the 1982 revised ACR criteria for the classification of SLE. Because of the referral pattern, it is not surprising that anti-dsDNA antibodies were found to be the best discriminator. In a more detailed analysis of the literature, Kavanaugh *et al.* ^18^ provided a more comprehensive guideline regarding the use of anti-DNA antibody tests in rheumatic disease. The ACR Ad Hoc Committee on Immunological Testing Guidelines chaired by Dr. A. F. Kavanaugh did a thorough evaluation of the literature. It came to the conclusion that the three commonly employed methods (i.e. ELISA, the Farr assay, and immunofluorescence using *Crithidia luciliae* as a substrate) correlated with each other when they were applied to populations of patients, but there were substantial discrepancies when these techniques were applied to individual patients. This conclusion may not be surprising, since each of these methods may detect different populations of anti-DNA antibodies due to affinities of the targeted antibodies for dsDNA and substrate differences. The Committee recommendation was that anti-DNA antibodies are useful in supporting the diagnosis of SLE in the setting of clinical presentation highly suggestive of the diagnosis. Although anti-DNA antibodies are rarely described in other rheumatologic conditions, a positive anti-DNA is not diagnostic of SLE but a negative test does not rule out the diagnosis. Regarding the correlation of anti-DNA antibodies with clinical activities, the Committee found that the correlation is modest at best. A similar conclusion was drawn regarding the correlation of anti-DNA antibodies with renal disease. It was also concluded that the presence of anti-DNA antibodies does not predict a flare of the disease. The Committee withheld its judgement regarding the usefulness of an increase in anti-dsDNA antibodies that may pre-date or may be associated with flare of disease activity because of lack of studies on this issue. These guidelines remain valid and useful more than a decade later.

Despite the ample amount of publications on anti-DNA antibodies in SLE, investigations on anti-DNA antibodies as related to SLE have continued. On the technical issue regarding different assays, a recent paper by Encosson *et al.* ^19^ compared IgG anti-dsDNA bead-base multiplex assay (FIDIS; Theradiag), fluoroenzyme-immunoassay (EliA; Phadia/Thermo Fisher Scientific), *Crithidia luciliae* immunofluorescence test (CLIFT; ImmunoConcepts) and line blot (EUROLINE; EUROIMMUN) on 187 patients with SLE, with patients with rheumatoid arthritis (RA) and progressive systemic sclerosis (pSS) as disease controls and healthy controls. It was shown that rare patients with RA and pSS were positive by the Crithidia immunofluorescence tests. The specificity of CLIFT in the authors’ laboratory was cited to be 98%. The other three assays were cited to have lower specificities. By adjusting the base line the other three tests achieved similar specificity to the CLIFT assay. In this population of SLE patients, the sensitivity of all four assays was in the low 20s. The stated conclusion was that “there is a great variability among anti-dsDNA assays and a stricter cut-off limit must be applied to acceptable SLE specificities of FIDIS, ELiA and EUROLINE”. These results were in accordance with those outlined in 18. They also suggest that the criterion for the inclusion of anti-dsDNA antibodies for the ACR classification of SLE should be modified similarly to the modification in the SLICC-12 (2012 Systemic Lupus International Collaborating Clinic Classification) criteria for SLE ^20^. The modification is that anti-dsDNA antibody levels should be above laboratory reference range (or twice the reference range if tested by ELISA).

The variability of different anti-dsDNA assays was highlighted in an article by Compagno *et al.* ^21^ published in the inaugural issue of Lupus Science & Medicine. The article described the clinical phenotype associated with various types of anti-dsDNA antibodies in patients with recent onset of rheumatic symptoms. 1073 patients were recruited from three academic centers in the three Scandinavian countries. 292 patients were found to be antinuclear antibody (ANA) positive. 292 patients were randomly selected from patients with negative ANA. These sera were assayed for anti-dsDNA antibodies by CLIFT at least three times by two commercial kits and four times by three solid phase ELISA kits. 37 patients dropped out. Of the 288 ANA-positive patients, 19.8% (n=57) carried the diagnosis of SLE. In contrast, only 2.3% (n=6) of the ANA-negative patients (n=259) were identified to have SLE. In the 288 ANA-positive sera, 39 (13.5%) sera were positive in any CLIFT and 50 (17.4%) were positive in any ELISA. Of the 259 ANA-negative sera, 20 (7.7%) were positive by any CLIFT and 49 (18.9%) were positive by any ELISA kits. There was low concordance between the CLIFT assays and ELISA assays with 25 CLIFT ^+^ELISA ^-^, 65 CLIFT ^-^ELISA ^+^, and 34 CLIFT ^+^ELISA ^+^. It was concluded that different anti-dsDNA antibodies are associated only modestly with nephropathy, pleuritis, alopecia, and lymphopenia.

The group of patients studied by Compangno *et al.* ^22^ was followed for a median of 4.8 years. The follow-up results were astonishing. It was concluded that CLIFT is not reliable as a diagnostic tool in unselected patients with rheumatic symptoms. CLIFT had low positive predictive value for SLE, in that only one out of 36 CLIFT positive patients who were not diagnosed with SLE at entrance developed SLE. Thus, for non-SLE patients, being CLIFT positive poses little risk of developing SLE within 5 years.

In the review by Mehra and Fritzler ^14^, it was concluded that the anti-chromatin/nucleosome antibodies may be superior to anti-dsDNA antibodies as a biomarker for SLE. In summary, this brief analysis of the literature supports the conclusion that anti-dsDNA antibodies are not specific or the best biomarker for SLE.

## A surge in anti-dsDNA antibody titer may not be a good predictor for either non-renal or renal flares in SLE

The data supporting the claim that rising titers of anti-dsDNA antibodies is a good predictor for flares in SLE have been reviewed in 2. Recently Pan *et al.* ^23^ published their retrospective study on the lupus cohort at the Hospital of Special Surgery in an attempt to correlate surges in anti-dsDNA antibodies with renal and non-renal flares. It was concluded that an anti-dsDNA surge was not predictive of renal flare. Regarding non-renal flares as measured by the SELENA-SLEDAI instrument, the data showed that an anti-dsDNA surge had a sensitivity of 62%, specificity of 80%, positive predictive value of 59%, and negative predictive value of 81% for a severe SELENA-SLEDAI flare. Although the authors concluded that a surge in anti-dsDNA titer predicts a severe SELENA-SLEDAI lupus flare within 6 months, the predictive value of such a surge appears to be, at best, of modest accuracy and clinical applicability.

Regarding the predictive value of a surge in anti-dsDNA antibody titer for a renal flare, this was investigated in 487 patients who had a history of lupus nephritis and an anti-dsDNA antibody titer ≥15 IU/ml at baseline (as measured by Farr assay) and who represented the treatment and placebo arms of patients being treated with a dsDNA-based bioconjugate, LJB394, in 2 clinical trial cohorts ^24^. The results ^24^ showed that “Changes in anti-dsDNA antibody levels were inversely correlated with changes in the C3 level ( *P*<0.0001 in both trials). Cox proportional hazards regression models showed that changes in anti-dsDNA antibody levels correlated with the risk of renal flare. The models predicted that a point estimate of a 50% reduction in anti-dsDNA antibody levels is associated with a 52% reduction (95% confidence interval [CI] 26–68%, nominal *P*=0.0007) and a 53% reduction (95% CI 33–69%, nominal *P*<0.0001) in the risk of renal flare in the 2 trials, respectively. In the 2 trials, the incidence of renal flare was lower in patients with sustained reductions in anti-dsDNA antibodies (3.0% and 4.1%, respectively) than in patients with stable or increasing antibody levels (21.3% and 20.3%, respectively)”. The results (that only ~20% of the patients with an anti-dsDNA antibody surge had a renal flare and that 3–4% of the patients without such a surge developed a renal flare) suggest that an anti-dsDNA antibody titer surge is, at best, of modest predictive value. The changes of anti-dsDNA antibody titers cannot be used in clinical practice to treat patients prophylactically, or to assure patients without an increase in anti-dsDNA antibodies that a renal flare will not occur.

## Anti-dsDNA antibodies may not be the antibodies that initiate lupus nephritis

Because of the initial report that anti-dsDNA antibodies were eluted from kidneys in patients with lupus nephritis, it has been suggested that these antibodies may initiate lupus nephritis. In the 1971 paper that proposed SLE to be a prototype of immune complex nephritis in man ^9^, Koffler *et al.* were able to demonstrate the concentration of antibodies to dsDNA in the kidney eluates from 5 out of 9 samples, suggesting that some of the cases studied may not have anti-dsDNA antibody deposits in the kidney. These authors also showed that other antigen-antibody complexes were deposited in the diseased kidneys. In a later paper ^10^, Koffler *et al.* stated that “acid buffer eluates were prepared from biopsy and two kidneys from necropsy which showed no histological or clinical evidence of renal disease. Immunofluorescent study of these tissues revealed a linear deposit of γG-globulin. The eluates obtained did not contain demonstrable anti-nuclear or anti-basement membrane antibodies”. The study by Mannik *et al.* ^25^ showed that antibodies of multiple specificities were eluted from the kidneys of patients who died of lupus nephritis. Many of the 25 renal eluates did not have anti-dsDNA antibodies. These findings suggest that anti-dsDNA antibodies may not be required for the pathogenesis of lupus nephritis. This hypothesis has been supported by our studies of the genetics of lupus nephritis in NZM2328 mice ^26, 27^. We found that a single locus on chromosome 4 controls the production of anti-dsDNA antibodies ^26^. The congenic strain NZM2328.C57L/Jc4 (NZM.L/Jc4) was generated by introgressing a genetic segment of chromosome 4 from C57L/J, a non-lupus prone strain where the gene controlling anti-dsDNA antibody production is located to NZM2328 ^27^. Female mice of NZM.L/Jc4 had little circulating ANA or anti-dsDNA antibodies. They developed immune complex-mediated nephritis with end-stage renal disease and early mortality in a manner similar to that of the parental strain NZM2328.

Recently, Bruschi *et al.* have published two papers relevant to this issue ^28, 29^. They eluted antibodies from 20 renal biopsy samples from patients with lupus nephritis. They identified 12 targeted podocyte molecules. It appears that α-enolase and annexin A1 were the most commonly targeted antigens ^28^. These patients have high titers of circulating antibodies to these two antigens and to dsDNA and C1q ^29^. Six of the 20 renal eluates had antibodies to α-enolase without antibodies to dsDNA, and 4 of the 20 eluates had antibodies to annexin A1 without anti-dsDNA antibodies. In these patients, anti-dsDNA antibodies did not play a role in the initiation of lupus nephritis.

## Concluding remarks

This review provides evidence that anti-dsDNA antibodies have a limited value in the diagnosis of SLE. These antibodies are useful in confirming the diagnosis in the clinical settings when SLE is likely to be the diagnosis. They have limited usefulness in monitoring disease activities and in predicting flares. In contrast to the current dogma that these antibodies may initiate lupus nephritis, they are not necessary or sufficient to cause lupus nephritis. It is likely that these antibodies play an amplification role in the pathogenesis of lupus nephritis, in that they interact with Toll-like receptors (TLRs) with subsequent release of type 1 interferons that amplify the autoantibody response. They may react with DNA released from podocytes undergoing apoptosis and implanted in the glomerular basement membrane, causing further renal damage *via* complement fixation and/or interaction with Fc receptors. These antibodies should be considered to be one of the many autoantibodies seen in SLE patients.

**Figure 1.**
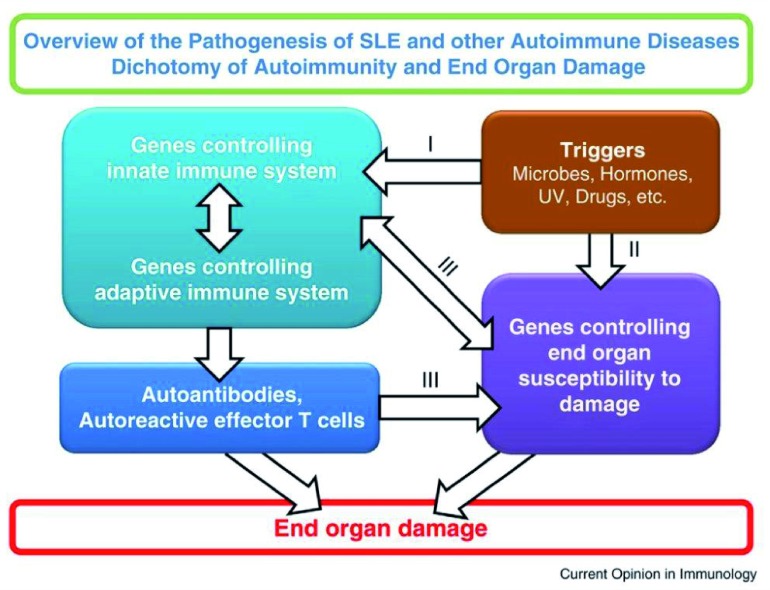
Interactive model for the pathogenesis of SLE. This model makes the assumption that environmental triggers act on susceptible hosts. The triggers act on both genes controlling immune responsiveness and genes for end organ damage. These are two independent yet interactive pathways. Pathway I leads to the generation of autoantibodies and autoreactive effector T cells. Pathway II provides autoantigens and/or soluble mediators that influence immune responsiveness. Pathways I and II interact at several levels as indicated by III. These interactions can lead to end organ damage. In this context, the end organ is the kidney and the autoimmune response is the production of autoantibodies to multiple autoantigens that form immune complexes to be deposited in the kidney ^32^.

Despite their limited value as biomarkers, anti-dsDNA antibodies are the most studied autoantibodies, and significant information has been generated regarding autoantibody formation and B cell development from studying these antibodies ^13, 30^. Undoubtedly further studies of these antibodies will yield significant information on B cell biology. They would also be useful in studying the interaction of autoantibodies with their targeted organs ^31^. Evidence has been accumulated to show that autoimmune diseases are the results of interactions of autoantibodies and autoreactive T cells with targeted organs. The targeted organs play an active role in this process. Autoimmunity and end organ damage are under separate genetic control ^32^. The model proposed by us as shown in Figure 1, for the pathogenesis of autoimmune diseases in general and SLE in particular, will be useful in placing the role of autoantibodies and autoreactive T cells in proper perspective. It will also explain the puzzling clinical observations that some SLE patients are serologically active and clinically quiescent while others are clinically active and serologically quiescent ^33, 34^.

In the review article by Isenberg *et al.* from 2007 ^2^, the authors asked, “Fifty years of anti-dsDNA antibodies: are we approaching journey’s end?”. Perhaps we can answer this question by saying that the journey should end regarding their role in the diagnosis of SLE and in their correlation with clinical activity and flares. Just as with many other autoantibodies, the events leading to the production of anti-dsDNA antibodies remain to be elucidated.

## Abbreviations

ACR, American College of Rheumatology; ANA, antinuclear antibody; CLIFT, *Crithidia luciliae* immunofluorescence test; LE, lupus erythematosus; pSS, progressive systemic sclerosis; RA, rheumatoid arthritis; SELENA, Safety of Estrogen in Lupus: National Assessment; SLE, systemic lupus erythematosus; SLEDAI, Systemic Lupus Erythematosus Disease Activity Index; SLICC-12, 2012 Systemic Lupus International Collaborating Clinic Classification.
