# Supplementary material for: Anti-dsDNA Antibodies are one of the many autoantibodies in systemic lupus erythematosus
Source: F1000Res. 2015 Oct 1;4(F1000 Faculty Rev):939. [Version 1] doi: 10.12688/f1000research.6875.1 (PMC4648223; doi:10.12688/f1000research.6875.1)
Supplement: Peer review history

# Peer review: Referee response for version 1

**DOI:** 10.5256/f1000research.7402.r10646

**Published:** 2015-10-01

**Authors:** Allan Gibofsky

**Affiliations:** 1 Hospital for Special Surgery, Weill Cornell Medical College, New York, NY, 10021, USA

I have read this submission. I believe that I have an appropriate level of expertise to confirm that it is of an acceptable scientific standard.

# Peer review: Referee response for version 1

**DOI:** 10.5256/f1000research.7402.r10645

**Published:** 2015-10-01

**Authors:** George Tsokos

**Affiliations:** 1 Center for Life Sciences, Beth Israel Deaconess Medical Center, Boston, MA, 02115, USA

I have read this submission. I believe that I have an appropriate level of expertise to confirm that it is of an acceptable scientific standard.
